# Supplementary figures and images for: Evaluation of the Abbott Panbio™ COVID-19 antigen detection rapid diagnostic test among healthcare workers in elderly care
Source: PLoS One. 2023 Feb 24;18(2):e0276244. doi: 10.1371/journal.pone.0276244 (PMC9955641; doi:10.1371/journal.pone.0276244)

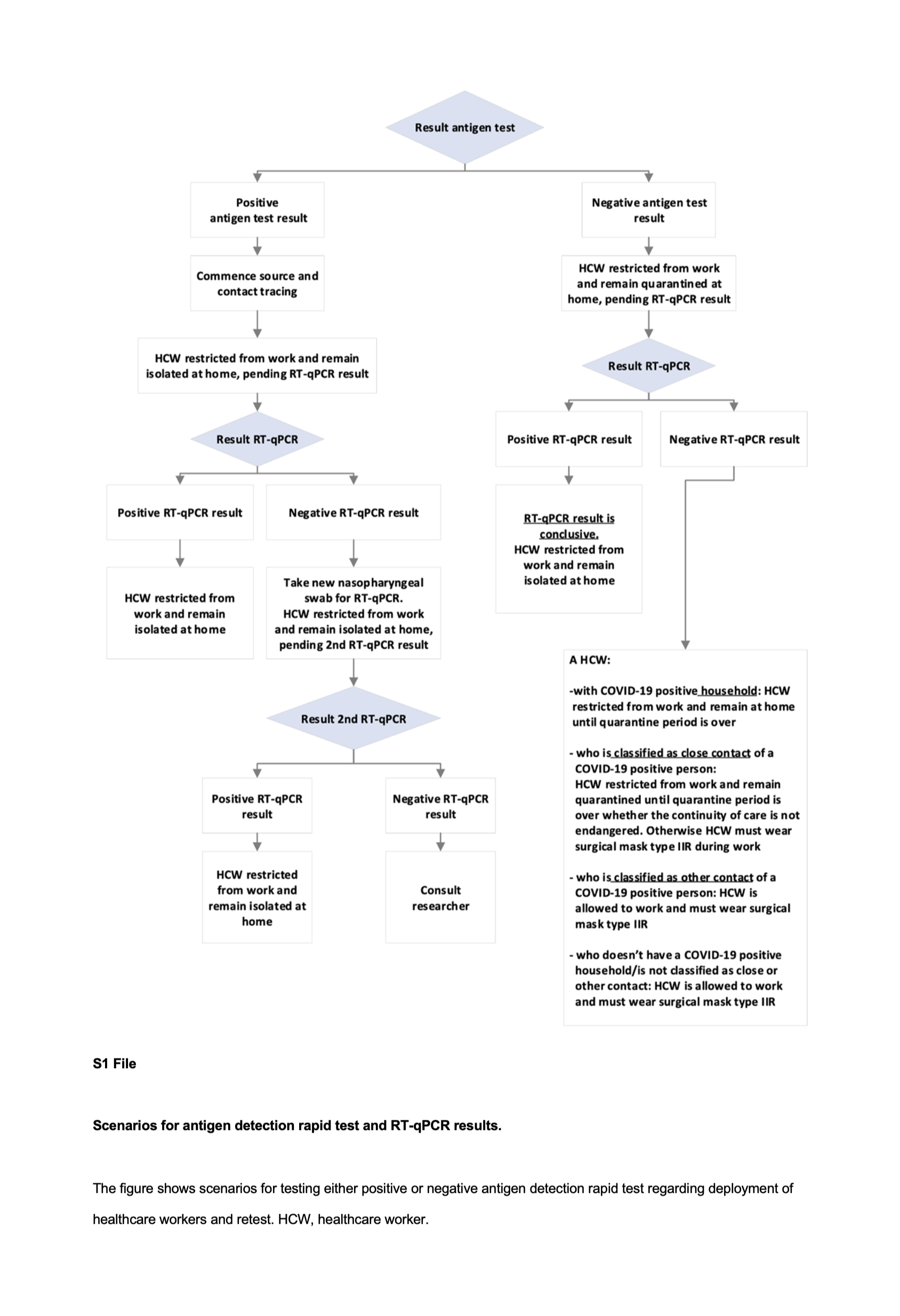

Supplement: S1 File — The figure shows scenarios for testing either positive or negative antigen detection rapid test regarding deployment of healthcare workers and retest. HCW, healthcare worker. (TIFF) [file pone.0276244.s001.tiff]
